# Mitotic count reflects prognosis of gallbladder cancer particularly among patients with T3 tumor

**DOI:** 10.3892/mco.2013.121

**Published:** 2013-05-16

**Authors:** KEITA KAI, MASANORI MASUDA, TAKAO IDE, YUKARI TAKASE, ATSUSHI MIYOSHI, KENJI KITAHARA, KOHJI MIYAZAKI, HIROKAZU NOSHIRO, OSAMU TOKUNAGA

**Affiliations:** 1 Departments of Pathology and Microbiology, Saga University, Faculty of Medicine, Saga City, Safa 849-8501, Japan; 2 Surgery, Saga University, Faculty of Medicine, Saga City, Safa 849-8501, Japan

**Keywords:** gallbladder cancer, mitotic counts, tumor infiltrative lymphocyte, p53, Ki-67 labeling index

## Abstract

The surgical strategy for gallbladder cancer (GBC) depends on the extent of the disease. Thus, the identification of useful prognostic markers exerting strong prognostic impact for each T stage would be beneficial in the development of rational therapeutic strategies. The purpose of this study was to identify useful prognostic markers of GBC for each T stage. CD8^+^ tumor-infiltrating lymphocytes (TIL), Ki-67 labeling index (LI), p53 nuclear expression and mitotic count (MC) were investigated as candidate prognostic markers. In total, 86 patients with invasive GBC were included. Of the prognostic markers examined, only MC showed a correlation with reduced survival (P=0.0383) in the univariate analysis of overall T stage. In the univariate analysis of T2 stage (n=31), only high p53 expression correlated with survival showing a positive correlation (P=0.0154). In the univariate analysis of T3 stage (n=40), the only factor showing a significant correlation with survival was MC (P=0.0113). Multivariate analysis, including N and M as factors, identified only MC as an independent prognostic factor in T3 stage GBC (P=0.0419). In conclusion, this study demonstrated the strong prognostic impact of MC in GBC, particularly in patients with T3 tumor.

## Introduction

The surgical strategy for gallbladder cancer (GBC) depends on the extent of the disease, particularly the T stage from the tumor, node, metastasis (TNM) classification (1). Identification of useful prognostic markers exerting a strong prognostic impact for each T stage would be beneficial in the development of rational therapeutic strategies for each T stage of GBC. Useful histological markers are well-known and easily evaluated in ordinary pathological examinations. This study therefore focused on CD8^+^ tumor-infiltrating lymphocytes (TIL), Ki-67 labeling index (LI), p53 nuclear expression and mitotic count (MC), all of which have been well investigated in other solid cancers as candidate prognostic markers in GBC.

CD8^+^ TIL have been considered as manifestations of host immune reactions against cancer cells and strong prognostic impact of CD8^+^ TIL has been found in a wide variety of solid cancer tissues (2–10). A gene on chromosome 10 encodes a nuclear protein of 345–395 kDa that is recognized by the antibody of the Ki-67 antigen. Ki-67 protein is expressed during the active phases of the cell cycle (G1, S, G2, and mitosis), but is absent from resting cells (G0). Ki-67 LI is thus considered a marker for cell proliferation and the prognostic impact of Ki-67 LI has been reported in various solid cancer tissues (11–14). p53 is a well-known tumor suppressor protein that is encoded by the TP53 gene, located on the short arm of chromosome 17. Mutations of the TP53 gene lead to loss of production of the normal p53 protein and synthesis of a mutated protein with an increased half-life which tends to accumulate in the nucleus and can be detected by immunohistochemical staining (15). The prognostic role of p53 nuclear expression as assessed by immunohistochemistry has been reported in various types of solid cancer (16–19). MC is widely recognized as an indicator of tumor malignancy and the prognostic impact of MC and classification or grading by MC status have been reported for various types of tumor (20–24).

The purpose of this study was to assess the prognostic impact of CD8^+^ TIL, Ki-67 LI, p53 nuclear expression and MC in GBC, according to T stage.

## Materials and methods

### Patients and staging

A total of 101 GBC patients underwent surgical treatment for the primary lesion at the Saga University Hospital between January, 1989 and December, 2011. Of these, 11 patients showing non-invasive intramucosal cancer and 4 patients for whom no tissue samples were preserved were excluded from the study. As a result, a final total of 86 patients with invasive GBC were enrolled in this study. Informed consent for the use of resected tissue was obtained from the patients, and the study protocol was approved by the Ethics Committee of the Faculty of Medicine at the Saga University. Clinical and histopathological staging were based on the TNM Classification of Malignant Tumors established by the International Union Against Cancer (7th edition, 2009) (1).

### Immunohistochemical staining and evaluation of MC

Sections cut from formalin-fixed paraffin-embedded tissue blocks were used. The primary antibodies used were CD8 (dilution 1:50, clone C8/144B; DakoCytomation, Glostrup, Denmark), p53 (prediluted, clone DO-7; Nichirei Biosciences, Tokyo, Japan) and Ki-67 (dilution 1:30, clone MIB-1; DakoCytomation). The slides were heated in ethylenediaminetetraacetic acid (EDTA) (pH 9.0) in a microwave oven for antigen retrieval. The EnVision™+ system (DakoCytomation) was used as the secondary antibody. Slides were visualized by diaminobenzidine tetrahydrochloride (DAB 4HCl) and nuclei were counterstained with hematoxylin. An Autostainer Plus^®^ automatic stainer (DakoCytomation) was used for staining. Ki-67 LI was determined using the ratio of positive nuclear staining of Ki-67 and classified as ≤10% (low group) or >10% (high group). Assessment of p53 was also determined by positive nuclear staining and classified as ≤30% (low group) or >30% (high group). The cut-off value of p53 and Ki-67 LI in previous studies varied greatly. The cut-off value of p53 and Ki-67 was determined to divide the cohort into two comparable groups effectively. CD8^+^ lymphocytes within the cancer cell nest were regarded as CD8^+^ TIL, according to a previous report (2). CD8^+^ TIL were counted in the three fields showing the most abundant distribution of CD8^+^ TIL using a ×10 objective lens. The number of CD8^+^ TIL was then determined as the mean count for these three fields. CD8^+^ TIL was analyzed separately on the tumor surface and the invasion front and was categorized as ≤10 (low group) and >10 (high group). Mitoses were counted in 10 high-power fields (HPF; magnification, ×400) on slides stained using hematoxylin and eosin (H&E) and categorized as ≤10/10 HPF or >10/10 HPF. Assessments of immunohistochemical staining and mitoses were performed and confirmed by consensus decision by two pathologists (M.M. and K.K. or Y.T. and K.K.).

### Statistical analysis

Statistical analysis was performed using the JMP software version 8 (SAS Institute, Cary, NC, USA). Statistical analysis to compare the two groups was performed using the Student’s t-test, the χ^2^ test and Fisher’s exact test, as appropriate. The survival analyses were performed as disease-specific survival, determined from the time of surgery to the time of cancer-related death or the most recent follow-up. The Cox proportional hazards model was applied for univariate and multivariate analyses. Postoperative survival curves were calculated using the Kaplan-Meier method. Differences in survival curves were compared using the log-rank test. P<0.05 was considered to indicate a statistically significant difference.

## Results

### Clinicopathological characteristics, status of CD8^+^ TIL, Ki-67 LI, p53 expression and MC and survival analysis for overall T stage

**Figure 1:**
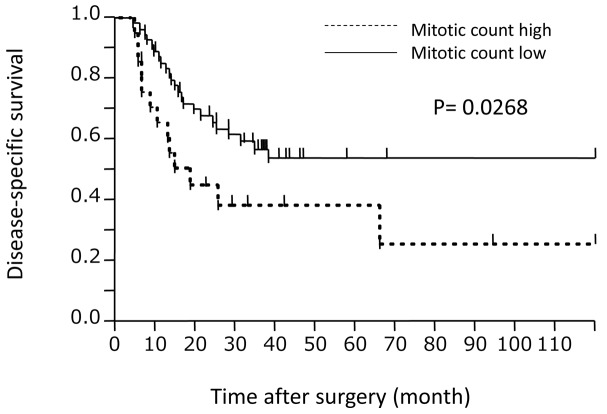
Kaplan-Meier curves for disease-specific survival according to mitotic counts in all stage patients (n=86). Mitotic count-high group (>10/10 HPF) showed significantly worse survival (P=0.0268, log-rank test). HPF, high-power field.

Patients comprised 27 men and 59 women, with a mean age of 68.8 years (range, 45–87 years) at the time of surgery. Nine patients (10.5%) were classified as T1b, 31 (36.0%) as T2, 40 (46.5%) as T3 and 6 (7.0%) as T4. Of the 86 patients, 68 (79.0%) were classified as the surface CD8^+^ TIL-high group and 18 (21.0%) as the surface CD8^+^ TIL-low group. Sixty-two patients (72.1%) were classified as the invasive site CD8^+^ TIL-high group and 24 (27.9%) as the invasive site CD8^+^ TIL-low group. Sixty patients (69.8%) were classified as the MC-low group and 26 (30.2%) as the MC-high group. Forty-two patients (48.8%) were classified as the Ki-67 LI-low group and 44 (51.2%) as the Ki-67 LI-high group. Fifty-seven patients (66.3%) patients were classified as the p53 expression-low group and 29 (33.7%) as the p53 expression high group. Uni- and multivariate analyses for disease-specific survival in the 86 GBC patients are shown in Table I. In the univariate analysis by Cox’s proportional hazards model, factors significantly correlated with survival were T, N and MC (P<0.0001, P<0.0001 and P=0.0383, respectively). The survival curve according to MC is shown in Fig. 1. The P-value calculated by log-rank testing was 0.0268. Multivariate analysis of the significant variables in the univariate analysis revealed only T and N as independent prognostic factors (P=0.005 and P=0.0113, respectively).

### Analysis in T2 patients

Univariate and multivariate analyses for disease-specific survival in T2 patients are shown in Table II. In the univariate analysis by Cox’s proportional hazards model, the only factors significantly correlated with survival were M and p53 expression (P=0.0311 and P=0.0154, respectively). Notably, the p53 expression-high group demonstrated significantly better outcomes compared with the low group. Multivariate analysis using significant variables from univariate analyses was conducted, but identified as independent prognostic factors.

### Analysis in T3 patients

**Figure 2:**
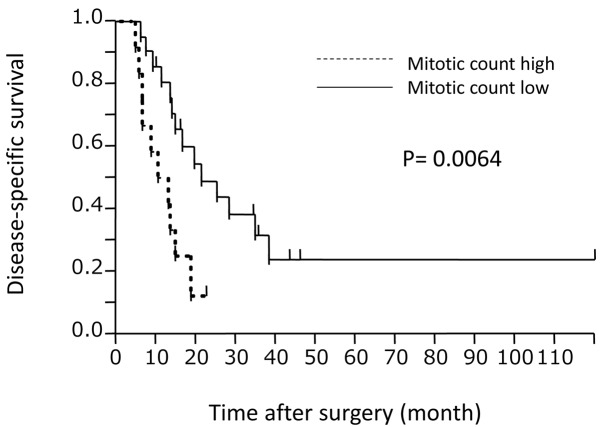
Kaplan-Meier curves with disease-specific survival according to mitotic counts in T3-stage patients (n=40). The group with high mitotic count (>10/10 HPF) showed significantly worse survival compared with the group with low mitotic count (P=0.0064, log-rank test); HPF, high-power field.

Univariate and multivariate analyses with disease-specific survival in T3 patients are shown in Table III. In the univariate analysis using Cox’s proportional hazards model, factors significantly correlated with survival were N, M and MC (P=0.017, P=0.0476 and P=0.0113, respectively). The survival curve according to MC is shown in Fig. 2. The P-value calculated by the log-rank test was 0.0064. Multivariate analysis using significant variables from the univariate analysis was conducted, identifying only MC as an independent prognostic factor (P=0.0419).

## Discussion

The prognosis following surgery for GBC is markedly different according to the results for the T stage, as are the therapeutic strategies (25,26). Thus, studies for GBC according to T stage better reflect actual prognosis after surgery and provide more useful information for clinical treatment compared with studies on overall GBC. Generally, survival of patients with T1 lesions is particularly good and simple cholecystectomy with or without lymphadenectomy is thus widely accepted as sufficient for T1 lesions (27). By contrast, survival of patients with T4 lesions is extremely poor and chemotherapy or palliative therapy is typically appropriate, except in rare cases where en bloc resection of multiple organs is applicable. This study therefore focused on patients with T2 and T3 tumors.

This study investigated correlations between survival after surgery and the status of CD8^+^ TIL, Ki-67 LI, p53 expression and MC as potential prognostic markers for GBC. However, results for these candidates were insufficient for use as markers, with the exception of the results for MC. Concerning CD8^+^ TIL, only one study that investigated the prognostic impact of CD8^+^ TIL, reporting that CD8^+^ TIL correlated with prolonged survival in univariate analysis was available (28). However, no prognostic impacts of surface or invasion front CD8^+^ TIL were observed in our cohort. Therefore, we consider the prognostic impact of CD8^+^ TIL in GBC to be controversial and suggest that further investigation is required. Several previous studies have reported no prognostic impact of p53 overexpression in GBC (29–33), whereas reports of poor prognosis with p53 overexpression are also available in the literature (34–36). Of note, the present study showed an association between p53 overexpression and favorable prognosis in T2 GBC. Taken together, the correlation between p53 and prognosis in GBC remains controversial, although gain of abnormalities in p53 protein is generally considered an early event in the progress of carcinogenesis and is likely to be the usual route for GBC development (37–40). A previous study reported that patients with GBC showing high Ki-67 exhibited worse postoperative prognosis compared with those showing low Ki-67 (41). Several previous studies reported that Ki-67 LI of cancer cells did not correlate with patient survival, supporting our results (30,32,33), which is in agreement with findings of the present study. The possible reason for Ki-67 not correlating with survival, despite the MC significant correlation of MC with survival, is that Ki-67 LI involves the G1, S and G2 phases of the cell cycle, while MC only involves the mitotic phase and might therefore reflect the rapidity of cell proliferation more sensitively compared with Ki-67 LI.

To the best of our knowledge, no previous studies have indicated the prognostic impact of MC in GBC and MC has not been applied in the histopathological diagnosis or grading of GBC. The degree of MC had a strong impact on survival in the present study. However, MC did not correlate with survival in patients with T2 tumor. This finding suggests that T2 GBC could be controlled by rational surgery involving regional lymphadenectomy and liver and bile duct resections (42) even in cases showing rapid growth with high MC tumor. By contrast, MC was identified as an independent prognostic factor in patients with T3 tumor. This suggests that tumors with rapid growth directly affect survival after surgery and that controlling rapidly growing tumors is difficult using a surgical approach alone. MC is easily evaluated by simple H&E staining. Additional investigation into MC in T3 GBC might contribute to information regarding prognosis after surgery as well as indications or selection of procedures for surgical treatment and/or adjuvant chemotherapy.

## Figures and Tables

**Table I:** Analysis of prognostic factors in 86 GBC patients.

		Univariate analysis		Multivariate analysis	
	
Characteristics	No.	HR (95% CI)	P-value	HR (95% CI)	P-value
T			<0.0001		0.0050
T1b, T2	40	1.00		1.00	
T3, T4	46	4.88 (2.35–11.17)		3.22 (1.41–8.00)	
N			<0.0001		0.0113
N0	41	1.00		1.00	
N1	45	6.39 (3.05–14.68)		3.21 (1.30–8.27)	
M			<0.0001		0.2407
M0	69	1.00		1.00	
M1	17	4.69 (2.30–9.30)		1.60 (0.73–3.53)	
Surface TIL			0.7485		
>10	68	1.00			
≤10	18	1.13 (0.54–2.67)			
Invasion front TIL			0.4719		
>10	62	1.00			
≤10	24	1.31 (0.64–2.96)			
Mitotic count			0.0383		0.1096
≤10/10 HPF	60	1.00		1.00	
>10/10 HPF	26	2.13 (1.04–4.15)		1.85 (0.87–3.85)	
Ki-67 LI			0.5191		
≤10%	42	1.00			
>10%	44	1.24 (0.64–2.46)			
p53 expression			0.9139		
≤30%	57	1.00			
>30%	29	0.96 (0.48–1.87)			

GBC, gallbladder cancer; HR, hazard ratio; CI, confidence interval; TIL, CD8^+^ tumor-infiltrating lymphocytes; LI, labeling index; HPF, high-power field; T, tumor; N, node; M, metastasis.

**Table II:** Analysis of prognostic factors in T2 GBC patients.

		Univariate analysis		Multivariate analysis	
	
Characteristic	No.	HR (95% CI)	P-value	HR (95% CI)	P-value
N			0.0504		
N0	21	1.00			
N1	10	3.80 (0.99–15.50)			
M			0.0311		0.1337
M0	28	1.00		1.00	
M1	3	5.79 (1.20–22.5)		2.32 (0.46–10.11)	
Surface TIL			0.5297		
>10	5	1.00			
≤10	26	1.86 (0.45–34.78)			
Invasion front TIL			0.4101		
>10	11	1.00			
≤10	20	1.87 (0.64–12.56)			
Mitotic counts			0.8558		
≤10/10 HPF	24	1.00			
>10/10 HPF	7	0.87 (0.13–3.59)			
Ki-67 LI			0.9994		
≤10%	11	1.00			
>10%	20	1.00 (0.26–4.76)			
p53 expression			0.0154		0.0623
≤30%	18	1.00		1.00	
>30%	13	0.13 (0.0070–0.71)		0.17 (0.0091–1.08)	

GBC, gallbladder cancer; HR, hazard ratio; CI, confidence interval; TIL, CD8^+^ tumor-infiltrating lymphocytes; LI, labeling index; N, node; M, metastasis; HPF, high-power field.

**Table III:** Analysis of prognostic factors in T3 GBC patients.

		Univariate analysis		Multivariate analysis	
	
Characteristics	n	HR (95% CI)	P-value	HR (95% CI)	P-value
N			0.0170		0.1209
N0	10	1.00		1.00	
N1	30	3.07 (1.21–9.42)		2.31 (0.81–7.52)	
M			0.0476		0.3942
M0	28	1.00		1.00	
M1	12	2.54 (1.01–6.22)		1.53 (0.57–4.09)	
Surface TIL			0.3747		
>10	7	1.00			
≤10	33	1.59 (0.60–5.49)			
Invasion front TIL			0.7769		
>10	10	1.00			
≤10	30	1.14 (0.47–3.18)			
Mitotic counts			0.0113		0.0419
≤10/10 HPF	23	1.00		1.00	
>10/10 HPF	17	3.30 (1.32–8.28)		2.66 (1.04–6.83)	
Ki-67 LI			0.1816		
≤10%	22	1.00			
>10%	18	1.75 (0.77–4.03)			
p53 expression			0.263		
≤30%	26	1.00			
>30%	14	1.59 (0.70–3.62)			

GBC, gallbladder cancer; HR, hazard ratio; CI, confidence interval; TIL, CD8-positive tumor infiltrating lymphocytes; HPF, high-power field; LI, labeling index; N, node; M, metastasis.

## References

[1] Sobin L, Gospodarowicz M, Wittekind C (2009). TNM Classification of Malignant Tumors.

[2] Naito Y, Saito K, Shiiba K, Ohuchi A, Saigenji K, Nagura H, Ohtani H (1998). CD8^+^T cells infiltrated within cancer cell nests as a prognostic factor in human colorectal cancer. Cancer Res.

[3] Guidoboni M, Gafà R, Viel A, Doglioni C, Russo A, Santini A, Del Tin L, Macrì E, Lanza G, Boiocchi M, Dolcetti R (2001). Microsatellite instability and high content of activated cytotoxic lymphocytes identify colon cancer patients with a favorable prognosis. Am J Pathol.

[4] Wakabayashi O, Yamazaki K, Oizumi S, Hommura F, Kinoshita I, Ogura S, Dosaka-Akita H, Nishimura M (2003). CD4^+^T cells in cancer stroma, not CD8^+^T cells in cancer cell nests, are associated with favorable prognosis in human non-small cell lung cancers. Cancer Sci.

[5] Prall F, Dührkop T, Weirich V, Ostwald C, Lenz P, Nizze H, Barten M (2004). Prognostic role of CD8^+^tumor-infiltrating lymphocytes in stage III colorectal cancer with and without microsatellite instability. Hum Pathol.

[6] Fukunaga A, Miyamoto M, Cho Y, Murakami S, Kawarada Y, Oshikiri T, Kato K, Kurokawa T, Suzuoki M, Nakakubo Y, Hiraoka K, Itoh T, Morikawa T, Okushiba S, Kondo S, Katoh H (2004). CD8^+^tumor-infiltrating lymphocytes together with CD4^+^tumor-infiltrating lymphocytes and dendritic cells improve the prognosis of patients with pancreatic adenocarcinoma. Pancreas.

[7] Zlobec I, Minoo P, Baumhoer D, Baker K, Terracciano L, Jass JR, Lugli A (2008). Multimarker phenotype predicts adverse survival in patients with lymph node-negative colorectal cancer. Cancer.

[8] Leffers N, Gooden MJ, de Jong RA, Hoogeboom BN, ten Hoor KA, Hollema H, Boezen HM, van der Zee AG, Daemen T, Nijman HW (2009). Prognostic significance of tumor-infiltrating T-lymphocytes in primary and metastatic lesions of advanced stage ovarian cancer. Cancer Immunol Immunother.

[9] de Jong RA, Leffers N, Boezen HM, ten Hoor KA, van der Zee AG, Hollema H, Nijman HW (2009). Presence of tumor-infiltrating lymphocytes is an independent prognostic factor in type I and II endometrial cancer. Gynecol Oncol.

[10] Shah W, Yan X, Jing L, Zhou Y, Chen H, Wang Y (2011). A reversed CD4/CD8 ratio of tumor-infiltrating lymphocytes and a high percentage of CD4(+) FOXP3(+) regulatory T cells are significantly associated with clinical outcome in squamous cell carcinoma of the cervix. Cell Mol Immunol.

[11] Seethala RR, Hunt JL, Baloch ZW, Livolsi VA, Leon Barnes E (2007). Adenoid cystic carcinoma with high-grade transformation: a report of 11 cases and a review of the literature. Am J Surg Pathol.

[12] Leuverink EM, Brennan BA, Crook ML, Doherty DA, Hammond IG, Ruba S, Stewart CJ (2008). Prognostic value of mitotic counts and Ki-67 immunoreactivity in adult-type granulosa cell tumour of the ovary. J Clin Pathol.

[13] Wrba F, Reiner A, Markis-Ritzinger E, Holzner JH, Reiner G, Spona J (1988). Prognostic significance of immunohistochemical parameters in breast carcinomas. Pathol Res Pract.

[14] Bruner JM (1994). Neuropathology of malignant gliomas. Semin Oncol.

[15] Greenblatt MS, Grollman AP, Harris CC (1996). Deletions and insertions in the p53 tumor suppressor gene in human cancers: confirmation of the DNA polymerase slippage/misalignment model. Cancer Res.

[16] Bosari S, Viale G, Radaelli U, Bossi P, Bonoldi E, Coggi G (1993). p53 accumulation in ovarian carcinomas and its prognostic implications. Hum Pathol.

[17] Fontanini G, Vignati S, Lucchi M, Mussi A, Calcinai A, Boldrini L, Chiné S, Silvestri V, Angeletti CA, Basolo F, Bevilacqua G (1997). Neoangiogenesis and p53 protein in lung cancer: their prognostic role and their relation with vascular endothelial growth factor (VEGF) expression. Br J Cancer.

[18] Assimakopoulos D, Kolettas E, Zagorianakou N, Evangelou A, Skevas A, Agnantis NJ (2000). Prognostic significance of p53 in the cancer of the larynx. Anticancer Res.

[19] Pancione M, Forte N, Fucci A, Sabatino L, Febbraro A, Di Blasi A, Daniele B, Parente D, Colantuoni V (2010). Prognostic role of beta-catenin and p53 expression in the metastatic progression of sporadic colorectal cancer. Hum Pathol.

[20] Sumithran E, Susil BJ, Looi LM (1988). The prognostic significance of grading in borderline mucinous tumors of the ovary. Hum Pathol.

[21] Elston CW, Ellis IO (1991). Pathological prognostic factors in breast cancer. I. The value of histological grade in breast cancer: experience from a large study with long-term follow-up. CW Elston and IO Ellis. Histopathology.

[22] Van Eeden S, Quaedvlieg PF, Taal BG, Offerhaus GJ, Lamers CB, Van Velthuysen ML (2002). Classification of low-grade neuroendocrine tumors of midgut and unknown origin. Hum Pathol.

[23] Kadota K, Suzuki K, Colovos C, Sima CS, Rusch VW, Travis WD, Adusumilli PS (2012). A nuclear grading system is a strong predictor of survival in epitheloid diffuse malignant pleural mesothelioma. Mod Pathol.

[24] Storr SJ, Safuan S, Mitra A, Elliott F, Walker C, Vasko MJ, Ho B, Cook M, Mohammed RA, Patel PM, Ellis IO, Newton-Bishop JA, Martin SG (2012). Objective assessment of blood and lymphatic vessel invasion and association with macrophage infiltration in cutaneous melanoma. Mod Pathol.

[25] Pilgrim C, Usatoff V, Evans PM (2009). A review of the surgical strategies for the management of gallbladder carcinoma based on T stage and growth type of the tumour. Eur J Surg Oncol.

[26] Mekeel KL, Hemming AW (2007). Surgical management of gall-bladder carcinoma: a review. J Gastrointest Surg.

[27] Wakai T, Shirai Y, Yokoyama N, Nagakura S, Watanabe H, Hatakeyama K (2001). Early gallbladder carcinoma does not warrant radical resection. Br J Surg.

[28] Nakakubo Y, Miyamoto M, Cho Y, Hida Y, Oshikiri T, Suzuoki M, Hiraoka K, Itoh T, Kondo S, Katoh H (2003). Clinical significance of immune cell infiltration within gallbladder cancer. Br J Cancer.

[29] Ajiki T, Onoyama H, Yamamoto M, Asaka K, Fujimori T, Maeda S, Saitoh Y (1996). p53 protein expression and prognosis in gallbladder carcinoma and premalignant lesions. Hepatogastroenterology.

[30] Hidalgo Grau LA, Badia JM, Salvador CA, Monsó TS, Canaleta JF, Nogués JM, Sala JS (2004). Gallbladder carcinoma: the role of p53 protein overexpression and Ki-67 antigen expression as prognostic markers.

[31] Kim YW, Huh SH, Park YK, Yoon TY, Lee SM, Hong SH (2001). Expression of the c-erb-B2 and p53 protein in gallbladder carcinomas. Oncol Rep.

[32] Jarnagin WR, Klimstra DS, Hezel M, Gonen M, Fong Y, Roggin K, Cymes K, DeMatteo RP, D’Angelica M, Blumgart LH, Singh B (2006). Differential cell cycle-regulatory protein expression in biliary tract adenocarcinoma: correlation with anatomic site, pathologic variables, and clinical outcome. J Clin Oncol.

[33] Kim WB, Han HJ, Lee HJ, Park SS, Song TJ, Kim HK (2009). Expression and clinical significance of cell cycle regulatory proteins in gallbladder and extrahepatic bile duct cancer. Ann Surg Oncol.

[34] Lee CS, Pirdas A (1995). p53 protein immunoreactivity in cancers of the gallbladder, extrahepatic bile ducts and ampulla of Vater. Pathology.

[35] Chang HJ, Yoo BC, Kim SW, Lee BL, Kim WH (2007). Significance of PML and p53 protein as molecular prognostic markers of gallbladder carcinomas. Pathol Oncol Res.

[36] Roa EI, Lantadilla HS, Ibacache SG, de Aretxabala UX (2009). p53 and p27 gene expression in subserosal gallbladder carcinoma. Rev Med Chil.

[37] Wistuba II, Gazdar AF, Roa I, Albores-Saavedra J (1996). p53 protein overexpression in gallbladder carcinoma and its precursor lesions: an immunohistochemical study. Hum Pathol.

[38] Kamel D, Pääkkö P, Nuorva K, Vähäkangas K, Soini Y (1993). p53 and c-erbB-2 protein expression in adenocarcinomas and epithelial dysplasias of the gall bladder. J Pathol.

[39] Itoi T, Watanabe H, Yoshida M, Ajioka Y, Nishikura K, Saito T (1997). Correlation of p53 protein expression with gene mutation in gall-bladder carcinomas. Pathol Int.

[40] Oohashi Y, Watanabe H, Ajioka Y, Hatakeyama K (1995). p53 immunostaining distinguishes malignant from benign lesions of the gall-bladder. Pathol Int.

[41] Shrestha ML, Miyake H, Kikutsuji T, Tashiro S (1998). Prognostic significance of Ki-67 and p53 antigen expression in carcinomas of bile duct and gallbladder. J Med Invest.

[42] Kohya N, Miyazaki K (2008). Hepatectomy of segment 4a and 5 combined with extra-hepatic bile duct resection for T2 and T3 gallbladder carcinoma. J Surg Oncol.

